# Supplementary material for: Modern Machine Learning as a Benchmark for Fitting Neural Responses
Source: Front Comput Neurosci. 2018 Jul 19;12:56. doi: 10.3389/fncom.2018.00056 (PMC6060269; doi:10.3389/fncom.2018.00056)
Supplement: Supplementary file 1 [file Data_Sheet_1.docx]

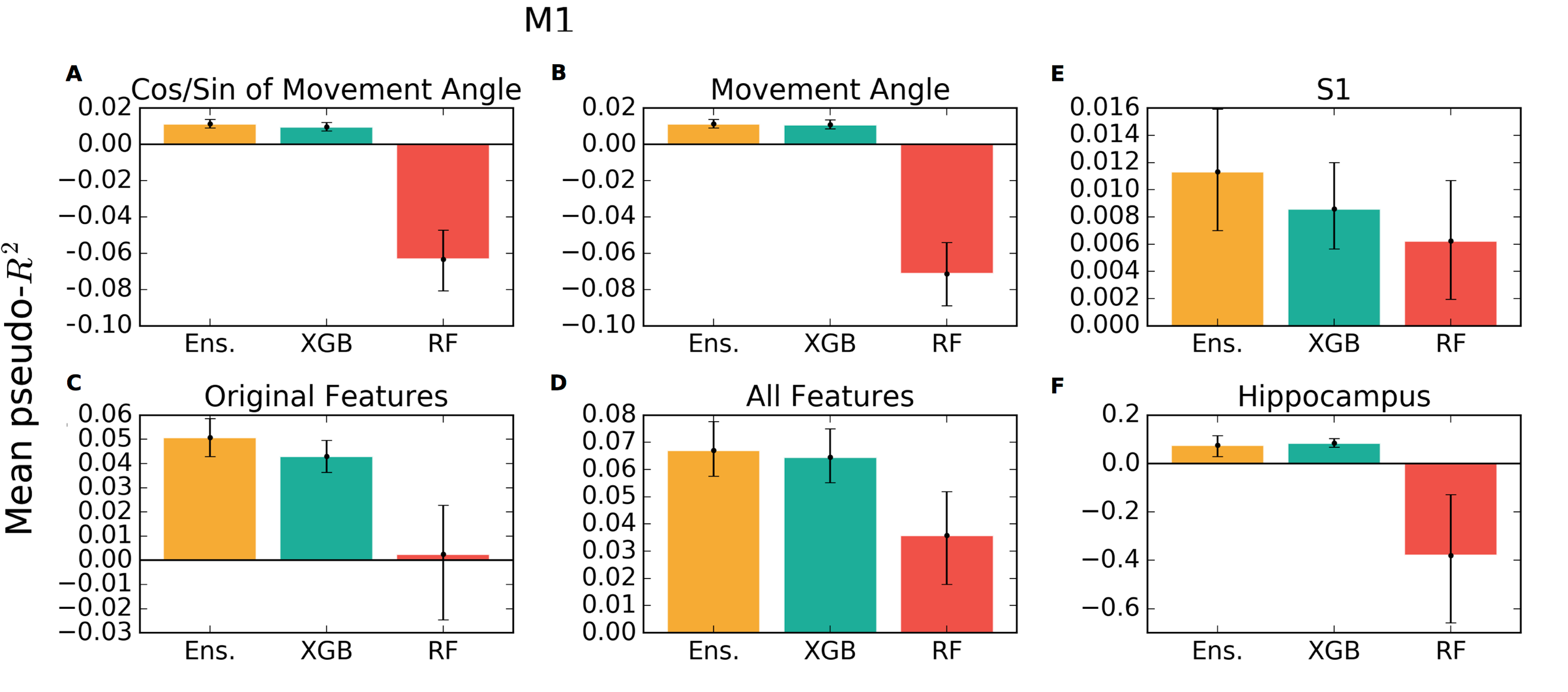


**Supplementary Figure 1:** The performance of the random forest (RF) method across all datasets and feature sets. Presented here is the mean pseudo-R^2^ score across the neural population, with the results of the ensemble and XGBoost shown again for comparison. This figure is presented in part to verify that the good performance of the ensemble does not stem directly from the RF. Error bars are the 95% bootstrapped confidence intervals. The M1 results are shown in **a-d,** the S1 dataset in **e**, and the hippocampus in **f.**


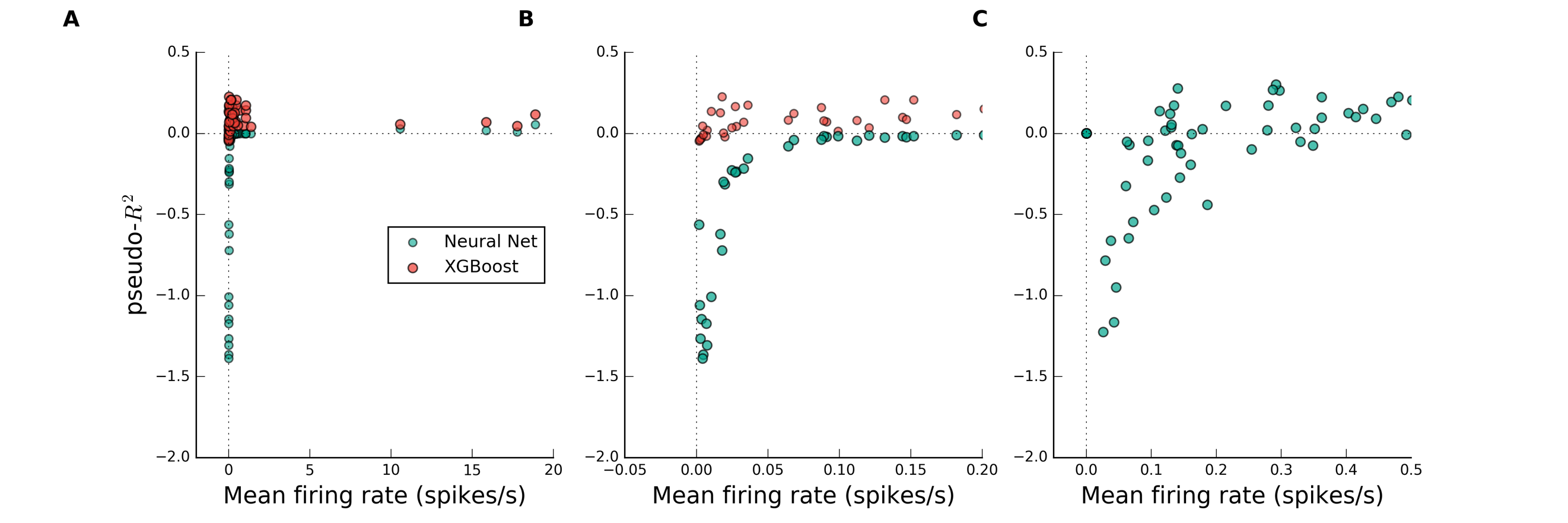
 **Supplementary Figure 2:** The performance of the neural network (NN) on the hippocampus dataset depended strongly on firing rate. **a**) The NN showed negative pseudo-R^2^ scores for all but the four highest-firing neurons. **b**) Same data as **(a)** but expanded x-axis, showing a very strong dependence of NN performance on neural firing rate. This decreasing curve is expected if the NN successfully predicted none of the plotted points; more misclassified examples (i.e. higher spike rate) lead to a lower pseudo-R^2^ score. More spiking examples will eventually lead to better fitting, causing the score to return upwards for higher rates as is observed (this return upwards is assisted by a relatively high elastic net regularization). This data was very sparse; a firing rate of 0.1 spikes/ second indicates that within the 110,925 time bins in the dataset, there were only 555 registered spikes. **c)** NN performance shows similar trends on GLM-simulated data of similar dataset size as a function of simulated firing rate. This points to firing rate as the likely cause of poor performance in the hippocampus dataset.


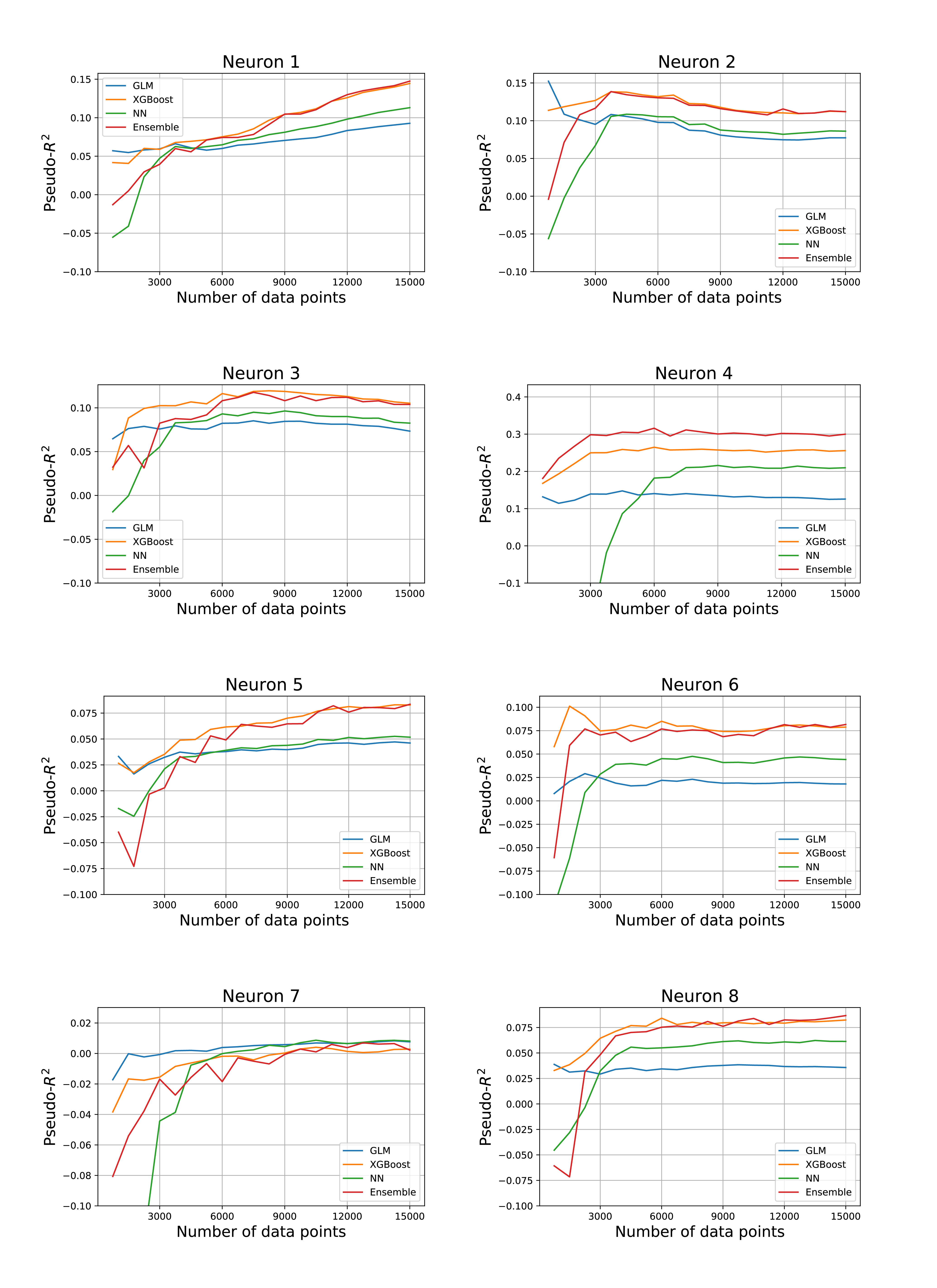


**Supplementary Figure 3** Convergence analyses for example neurons of the M1 dataset. Each of the four methods were fit to a fraction of the data and scored on the same test data. For some neurons, the GLM performed best at small data sizes. For others, such as neurons 4 and 6, the GLM underperformed other methods for all fractions of training data. The neural network was most sensitive to small data sizes. Interestingly, the ensemble was not always the best method at small data sizes. We suspect this is because the ensemble was chosen to be optimal for the full dataset. All hyperparameter choices were chosen to be the same as for the full dataset (as performing a new search at each fraction for each neuron was prohibitively computationally expensive).
